# Facilitators of co-leadership for quality care

**DOI:** 10.1136/bmj-2022-071330

**Published:** 2023-05-05

**Authors:** Olive Cocoman, Martin Dohlsten, Ernest Konadu Asiedu, Desalegn Bekele Taye, Margaret Mannah, Bongani Chiikpulo, Nana Mensah Abramah, Isabella Sagoe-Moses

**Affiliations:** 1 Quality of Care Network Secretariat, Department of Maternal, Newborn, Child and Adolescent Health and Ageing, World Health Organization, Geneva, Switzerland; 2 Department of Maternal, Newborn, Child and Adolescent Health and Ageing, World Health Organization, Geneva, Switzerland; 3 National Centre for Coordination for Early Warning and Response Mechanisms, Ministry of Health, Accra, Ghana; 4 Federal Ministry of Health, Addis Ababa, Ethiopia; 5 Department of Reproductive, Maternal, Newborn, and Child Health, Ministry of Health and Sanitation, Freetown, Sierra Leone; 6 Quality Management Directorate, Ministry of Health, Malawi; 7 London School of Hygiene and Tropical Medicine, London, UK; 8 Ghana Health Service, Accra, Ghana

## Abstract

**Olive Cocoman and colleagues** argue that national leadership for quality of care requires working in a co-leadership model such that quality and programme units have equal standing and clearly defined individual roles and responsibilities

Leadership is key to delivering health system change, including to improve quality of care.^1^ National health leadership is responsible for setting a country’s vision and strategy for health and to bring stakeholders together to implement that strategy.^2^ Ministries of health face multiple challenges to effective leadership, including political pressures, frequent personnel changes, budget limitations or cuts, and the personal efficacy of the leaders involved. Strategic approaches, implementation paths, and resource allocation also vary, giving rise to different strengths or stresses across health systems.

The World Health Organization describes national leadership as underpinning all efforts to improve quality of care.^2^ Leadership is essential for developing policies and strategies on quality or care and deploying the necessary resources (human, technical, and financial) to implement those strategies. Despite the importance of national leadership, most attempts to improve quality of care in low and middle income countries over the past two decades have been externally driven delivered by partners at the “micro” or facility level. 

In the absence of national leadership driving improvement in quality of health services, quality of care remains a “project” rather than being integrated into the health system as a core function.^3^ Leadership is not always linear and may be difficult to assign or negotiate when multiple health programmes or stakeholders have a joint mandate to improve quality of care. Indeed, the strengths of national and subnational quality of care structures vary widely across countries.^4^

Learning from the 11 countries in the Network for Improving Quality Care for Maternal, Newborn and Child Health (Quality of Care Network) suggests that the strength of national and subnational leadership structures depends on achieving successful co-leadership between government units designated to work on quality of care and programme units dedicated to specific health areas such as HIV or non-communicable diseases. Co-leadership occurs when two or more leaders at equal seniority share responsibilities for leadership on a specific effort.^5^ Drawing on the experience of the Quality of Care Network we suggest how countries can make the model work.

## Co-leadership can be challenging to implement

With growing evidence of the impact and cost of poor care, in 2018, WHO, the World Bank, and the Organisation for Economic Cooperation and Development called for all ministries of health to develop a national policy and strategy for quality of care at national, subnational, and facility levels.^6^ ^7^ ^8^ Twenty five countries across all continents have since implemented a national strategy, six of which are part of the Quality of Care Network (box 1).^13^ One of the first steps in the process is to establish a quality unit in the ministry of health to develop and implement the national quality strategy. The quality unit decides on the content of national policies and strategies and how to monitor to progress on quality of care at national, subnational, district, and facility level as well as coordinating and aligning the multiple stakeholders, including professional bodies, insurance agencies, institutional boards, and subnational, district, and facility teams to drive quality improvement.^15^ ^16^

Box 1 **The Quality of Care Network**

The Network for Improving Quality Care for Maternal, Newborn and Child Health (Quality of Care Network) brings together the individuals responsible for leading maternal, newborn, and child health (MNCH) and quality improvement within the health ministries of 11 countries: Bangladesh, Cote D’Ivoire, Ethiopia, Ghana, India, Kenya, Malawi, Nigeria, Sierra Leone, Tanzania, and Uganda.^9^ These countries have adopted and implemented WHO technical guidance on improving quality of maternal, newborn, and child care in health facilities.^10^ ^11^ As part of a roadmap for progress, two leadership outputs were agreed in 2017. By early 2023 all 11 had put in place national and sub-national governance structures and developed a costed national plan for improving quality of care.^12^

The network is delivering learning from both successes and failures on how national institutions can improve quality of care through continual monitoring of implementation processes and impact and outcome data.^9^ ^12^ ^13^ ^14^ Learning is being documented in national and subnational learning forums so it can be distilled and shared within and across the network.

Quality of care is also affected by the decisions of units delivering specific health programmes on resourcing, service delivery, and accountability.^17^ Therefore, the quality unit leadership must work collaboratively with programme units, which also have responsibility for implementing the national quality strategy. Additionally, the quality unit ensures coherence of quality of care activities across programmes, aiming to break any existing fragmentation of activities, building a consolidated quality of care agenda across programmes and provide unified oversight to reduce vertical programming.^17^

Multiple challenges exist when establishing co-leadership structures. One critical barrier to progress is how well the leaders responsible for quality of care work together with different budget lines, levels of resource, areas of expertise, and stakeholders and accountabilities. At national and subnational levels, it may be challenging to prevent confusion on roles or responsibilities or to negotiate competition for resources between the team working under the quality unit and the teams working under programmatic leads, even when there are shared goals around quality improvement and health outcomes.^4^ ^15^ In practice, one unit may overpower the other.^4^ Another risk is that one or more units may be unwilling or unable to work collaboratively, resulting in fragmentation or verticalisation of efforts.^4^ Communication breakdowns or other failures in co-leadership between quality and programme units mean that work on specific health areas may not advance and programmes may miss out on valuable guidance from quality leadership.^4^

## Organisational structure matters

Ensuring that quality and programme units have equal standing in the health ministry is important to overcoming barriers to effective co-leadership. Ethiopia provides a clear example of the way in which the organisational structure of a national ministry of health can limit the ability of quality units and programme units to achieve a co-leadership. In 2016, Ethiopia’s national quality strategy and its operational plan were developed by a new quality unit, the Health Service Quality Directorate.^18^ Regional health bureaus were assigned to develop operational plans for each region,^18^ and maternal, newborn, and child health (MNCH) was used as the first programme of joint work. Efforts to build capacity among health workers were jointly supervised by quality of care leads and MNCH programme leads, who established a robust facility and district learning system for improving quality of care.^19^ As such, the implementation of the national quality strategy was co-led by a national healthcare quality steering group comprising the directorate and programme leads.

However, programme leads reported to the state minister of health, whereas the directorate reported to the Medical Services Unit, which in turn reported to the state minister of health. The difference in seniority between the directorate and programme leads generated administrative and communication barriers,^20^ and the steering group was unable to coordinate between the quality and programme units.^20^ The progress review of the national quality strategy in 2020-21 concluded that the efficient use of resources and support for quality were negatively affected by the unequal organisational structure.^21^ ^22^ A new organisational structure has been devised, and the Health Service Quality Directorate has been reconfigured into a larger unit that will report directly to the minister of health, thus creating a parallel hierarchy.^23^

Similarly, in Malawi, the Ministry of Health aimed to address verticalisation and fragmentation from various quality of care initiatives by establishing a quality improvement unit in the Directorate of Policy and Planning in 2015. By the end of 2016, however, issues with hierarchy like those experienced in Ethiopia, made it necessary to elevate the small unit to a directorate, with equal standing to programme directorates.^24^

Sierra Leone is at an earlier stage in developing a co-leadership model. Quality of care policy and strategies have rapidly advanced since 2019, and quality standards for MNCH were used as the entry point. A national quality manager sits in the Department for Reproductive and Child Health, and while national quality MNCH roadmaps have been developed, no quality of care plans have been developed for other areas of healthcare.^25^ Learning from other network countries, and Ethiopia in particular, suggests that the next step should be to establish a national quality unit at the same organisational level as other health programme units, which will create a national quality and programme co-leadership model relevant to all health programmes. 

## Clear roles and responsibilities must be established

In addition to organisation of ministries of health to put quality units on the same level as other health programme units, co-leadership depends on clearly defining the roles and responsibilities of the different leads. Shared responsibility by the quality management unit at the Ministry of Health and the MNCH programme unit in Ghana has been key to progress. Ghana’s health system is pluralistic: the Ministry of Health develops policies, mobilises resources, and evaluates programmes and projects. Ghana Health Service (GHS) is the public services implementing agency with organisational structures at national, regional, and district levels. The quality management unit developed a national healthcare quality strategy in 2017.^26^ GHS developed guidelines for the implementation of the strategy, which prioritised maternal and child health.^27^ Verticalisation and fragmentation were avoided by carefully defining and upholding the leadership roles and responsibilities at national, regional, and district levels, including the facilities implementing the strategy.

In 2018, for example, a national MNCH quality of care technical working group was established to provide overall leadership. The head of the quality management unit and the deputy director of the family health programme in GHS were appointed co-chairs to lead and jointly guide the use of resources to implement the quality strategy.^28^ All roles and responsibilities were defined in the guidelines for implementation. Regional quality and safety management teams were established in all 16 regions, with each responsible for planning, supervising, monitoring, and evaluating the implementation of quality and patient safety programmes within their region in collaboration with programme quality teams. The regional teams ensure that agreed plans are completed successfully and supervise the establishment of programme quality teams in all districts and facilities.^27^ The national technical working group allocates funding to all regional teams, aligning resources behind a common agenda. The clarity of structures and roles at national and regional level established clear entry points for implementation partners to support individual regions as required. Based on the efficacy of this model, donors supported financial resource gaps in eight regions. As a result, by 2022, all 16 regions were supported to scale up quality maternal and child healthcare.^29^

Malawi’s experience corroborates the importance of defining roles and responsibilities for effective co-leadership. In 2017, Malawi’s Quality Management Directorate launched the quality management policy and strategy (2017-2022) and national MNCH quality of care roadmap (2017-2021). As in Ghana and Sierra Leone, MNCH was the entry point for improving quality of healthcare, and this is co-led by the Quality Management Directorate and Reproductive Health Department.^30^ A steering committee was established and is co-led by the quality and programme leads. However, the responsibilities of the directorate and the and Reproductive Health Department were not defined and agreed on, and this resulted in a lack of clarity regarding how or by whom collaboration would be built with district leadership and with other relevant health departments, such as the community health and nursing and clinical departments. The lack of clarity on responsibilities incurred delays, with periods of inaction on quality of care until 2019 when the organisational structure for the interface between national and district levels was completed and communicated to districts.^31^ ^32^ Learning from this experience has informed the subsequent phase of work, and roles and responsibilities have been carefully defined in the current national health strategic plan.^31^ ^33^

## Learning the lessons

Our examples show how lack of adequate organisational structures for quality and poorly defined roles and responsibilities result in loss of time, human, and financial resources for quality of care, and a loss of support for quality. Establishing organisational structures that enable co-leadership between quality and programme leadership are increasingly recognised as critical for successful quality of care. As countries in the Quality of Care Network continue to learn from practice and experience, engage in frequent review of their processes and outcomes, and use this knowledge to inform improvements in practice, this implementation feedback cycle should guide leaders’ decision making within and between countries.

**Key messages**

- Country efforts to improve quality of care require joint leadership from national quality departments and specific health programmes
- Experience from a network of countries suggests that establishing organisational structures so that leaders have equal influence
- Clarifying roles and responsibilities is also essential to support effective co-leadership
- Conversely, weaknesses in co-leadership incur losses of time, investments, and support for quality

## Notes

Contributors and sources: This article arose from a meeting of ministries of health and partners from countries in the Network for Improving the Quality of Care for Maternal, Newborn and Child Health, over three days in Accra, Ghana, in March 2023 to discuss progress and barriers to realising quality MNCH. Five authors on this paper are the programme or quality unit leads from ministries of health and have or are currently leading the work described. The views expressed are those of the authors and do not necessarily reflect those of WHO.

Provenance and peer review: Commissioned; externally peer reviewed.

This article is part of a collection proposed by the World Health Organization and the World Bank and commissioned by *The BMJ*. *The BMJ* peer reviewed, edited, and made the decision to publish these articles. Article handling fees are funded by the Bill and Melinda Gates Foundation. Jennifer Rasanathan, Juan Franco, and Emma Veitch edited this collection for *The BMJ*. Regina Kamoga was the patient editor.

## References

[1] World Health Organization. Everybody's business: strengthening health systems to improve health outcomes: WHO's framework for action. 2007. https://apps.who.int/iris/handle/10665/43918

[2] World Health Organization. Quality in primary health care. 2018 https://www.who.int/publications/i/item/WHO-HIS-SDS-2018.54

[3] Waiswa P . Institutionalization of projects into districts in low-and middle-income countries needs stewardship, autonomy, and resources. Glob Health Sci Pract 2020;8:144-6. 10.9745/GHSP-D-20-00170. 32614779 PMC7326513

[4] World Health Organization . The network for improving quality of care for maternal, newborn and child health: evolution, implementation and progress. 2017-2020 report. World Health Organization, 2021.

[5] Hennan D Bennis W . Co-leaders; the power of great partnerships. Wiley, 1999.

[6] Kruk ME Gage AD Joseph NT Danaei G García-Saisó S Salomon JA . Mortality due to low-quality health systems in the universal health coverage era: a systematic analysis of amenable deaths in 137 countries. Lancet 2018;392:2203-12. 10.1016/S0140-6736(18)31668-4. 30195398 PMC6238021

[7] World Health Organization, Organisation for Economic Co-operation and Development, World Bank. Delivering quality health services: a global imperative for universal health coverage. 2018. https://apps.who.int/iris/handle/10665/272465

[8] Syed SB Leatherman S Mensah-Abrampah N Neilson M Kelley E . Improving the quality of health care across the health system. Bull World Health Organ 2018;96:799. 10.2471/BLT.18.226266. 30505024 PMC6249706

[9] World Health Organization. Guidance on developing national learning health-care systems to sustain and scale up delivery of quality maternal, newborn and child health care. 2022. https://apps.who.int/iris/handle/10665/353739.

[10] World Health Organization. Standards for improving quality of maternal and newborn care in health facilities. 2016. https://apps.who.int/iris/bitstream/handle/10665/249155/9789241511216-eng.pdf?sequence=1.

[11] World Health Organization. *Leadership for quality: a brief on progress and learning from implementation 2017-2020* *.* *2021* *.* https://www.qualityofcarenetwork.org/knowledge-library/leadership-quality-brief-progress-and-learning-implementation-2017-2020

[12] World Health Organization. Network for Improving the Quality of Care for Maternal, Newborn and Child Health. Goals. https://www.qualityofcarenetwork.org/about#goals

[13] WHO. *Report of the first global meeting of the Network for Improving Quality of Care for Maternal, Newborn and Child Health in Lilongwe, Malawi, WHO February* *2017.* https://www.qualityofcarenetwork.org/knowledge-library/report-first-global-meeting-network-improving-quality-care-maternal-newborn-and

[14] World Health Organization*. Improving the quality of care for maternal, newborn and child health: implementation guide for national, district and facility levels. 2022* *.* https://www.qualityofcarenetwork.org/knowledge-library/improving-quality-care-maternal-newborn-and-child-health-implementation-guide)

[15] Leatherman S Sutherland K . Designing national quality reforms: a framework for action. Int J Qual Health Care 2007;19:334-40. 10.1093/intqhc/mzm049. 17947385

[16] WHO. Handbook for national quality policy and strategy. a practical approach for developing policy and strategy to improve quality of care. 2018. https://www.who.int/publications/i/item/9789241565561

[17] Kruk ME Gage AD Arsenault C . High-quality health systems in the Sustainable Development Goals era: time for a revolution. Lancet Glob Health 2018;6:e1196-252. 30196093 10.1016/S2214-109X(18)30386-3 PMC7734391

[18] Federal Democratic Republic of Ethiopia, Ministry of Health. Ethiopian national health care strategy, 2016-2020. Transforming the quality of health care in Ethiopia., http://repository.iifphc.org/handle/123456789/737?show=full

[19] Quality of Care Network. Ethiopia country page. https://www.qualityofcarenetwork.org/country-data/ethiopia *)*

[20] Bekele Taye D. Presentation at Improving Quality of Nutrition MNCH Services: Global resources, opportunities & lessons from Ethiopia, 22 Mar *2022* *.* https://www.childhealthtaskforce.org/events/2022/03/improving-quality-nutrition-mnch-services-global-resources-opportunities-lessons

[21] Federal Democratic Republic of Ethiopia, Ministry of Health. National health care quality strategy review. Ministry of Health, 2020.

[22] Federal Ministry of Health, Ethiopia. National Health Care Quality and Safety Strategy 2021-2025. *2021* https://e-library.moh.gov.et/library/index.php/moh-resource-library/?wpv-thematic-area=health-care-quality&wpv_aux_current_post_id=1677&wpv_aux_parent_post_id=1677&wpv_view_count=1678

[23] Federal Ministry of Health Ethiopia. Organizational structure 2022, https://www.moh.gov.et/site/Organizational_Structure

[24] Ministry of Health Malawi, WHO. Learning from quality of care leadership practice. Global Meeting of the Network for Improving the Quality of Care for MNCH. Accra, Ghana, Mar 2023. https://www.qualityofcarenetwork.org/knowledge-library?title=poster+malawi

[25] *Ministry of Health and Sanitation. National learning forum for quality maternal and newborn health, Sierra Leone* *.* *2021* https://www.qualityofcarenetwork.org/country-data/sierraleone

[26] Ministry of Health Ghana. *National health quality strategy (2017-2021).* Ministry of Health Ghana, 2016. https://www.moh.gov.gh/wp-content/uploads/2017/06/National20Quality20Strategy20Ghana.pdf

[27] Ghana Health Service . Guidelines for the implementation of the national quality strategy. Ghana Health Service, 2019.

[28] Ministry of Health Ghana. Launch of WHO implementation guidance for improving the quality of maternal, newborn and child health, September 6 2022 https://www.qualityofcarenetwork.org/webinars/series-2-webinar-17-launch-qoc-mnch-implementation-guide

[29] National Learning Forum for Quality Maternal and Newborn Health, Ministry of Health Ghana and Ghana Health Services, September . 2022 https://www.qualityofcarenetwork.org/country-data/ghana

[30] Ministry of Health Malawi. National MNCH quality of care roadmap (2017-2021).

[31] First National Quality of Care Conference, Lilongwe, Malawi, October 2022 and webinar https://www.qualityofcarenetwork.org/webinars/series-7-webinar-4-malawi-national-learning-forum

[32] Shawar Y, Djellouli N, Kohenour A, et al. Factors shaping network emergence: a cross-country comparison of quality of care networks in Bangladesh, Ethiopia, Malawi and Uganda. MedRxiv 2023. https://www.medrxiv.org/content/10.1101/2023.03.29.23287925v1.full.pdf 10.1371/journal.pgph.0001839 PMC11265678 39042649

[33] Ministry of Health Malawi. National Quality of Care Standards 2022.

