# Facilitators of co-leadership for quality care

**DOI:** 10.1136/bmj.p2904

**Published:** 2023-12-07

**Facilitators of co-leadership for quality care**

Bongani Chikwapulo’s name was misspelt in this article by Olive Cocoman and colleagues (BMJ 2023;381:e071330, doi:10.1136/bmj-2022-071330). The online version has been corrected.

